# The effects of feeding increasing levels of added fat to growing-finishing pigs when fed with or without narasin (Skycis)

**DOI:** 10.1093/tas/txaf088

**Published:** 2025-07-12

**Authors:** Kelsey L Kyle, Dustin D Boler, Clayton S Chastain, Eric Parr, Jorge Estrada, Danielle C Johnson, Casey Neill, Jonathan T Baker, Michael W Welch

**Affiliations:** Carthage Veterinary Service Ltd., Carthage, IL 62321, USA; Carthage Veterinary Service Ltd., Carthage, IL 62321, USA; Carthage Veterinary Service Ltd., Carthage, IL 62321, USA; Carthage Veterinary Service Ltd., Carthage, IL 62321, USA; Carthage Veterinary Service Ltd., Carthage, IL 62321, USA; Carthage Veterinary Service Ltd., Carthage, IL 62321, USA; Carthage Veterinary Service Ltd., Carthage, IL 62321, USA; Carthage Veterinary Service Ltd., Carthage, IL 62321, USA; Carthage Veterinary Service Ltd., Carthage, IL 62321, USA

**Keywords:** energy, fat, growing-finishing, narasin, pig, Skycis

## Abstract

The objective was to evaluate the effects of feeding increasing energy by increasing fat (corn oil) levels to growing-finishing pigs when fed with or without narasin (Skycis; Elanco Animal Health, Greenfield, IN). A total of 2,194 pigs with an initial body weight of 35.6 ± 3.6 kg were housed in 88 mixed-sex pens (25 pigs/pen). Each treatment combination was replicated 11 times. Pigs were fed in a 4 × 2 factorial arrangement of treatments in a randomized complete block design. Factors included added fat level (0.0%, 1.3%, 2.6%, or 4.0%) and narasin (0 mg/kg or 15 mg/kg). Pigs were provided ad libitum access to feed and water throughout the study and weighed on day 0 (start of experimental feeding period), 30, 54, and 80. Pigs were marketed over the course of 4 wk with the heaviest pigs removed during each marketing event. There were significant interactions between narasin and energy on overall grow-finish average daily gain (ADG), average daily feed intake (ADFI), and gain-to-feed (G:F) (P ≤ 0.05). Pigs that were fed 0% added fat and 15 mg/kg narasin gained 0.03 to 0.04 fewer kg per day (*P* ≤ 0.05) compared to pigs fed 2.6% added fat and 15 mg/kg narasin and pigs fed 4% added fat with narasin or no narasin. Pigs fed 0% added fat and no narasin ate at least 0.10 more kg per day (*P* ≤ 0.03) compared to all other treatments. Pigs fed 0% added fat and no narasin had the lowest (*P* ≤ 0.01) gain to feed (G:F) by at least 0.009 compared to all other treatments. The G:F of pigs fed 0 mg/kg narasin increased (*P* ≤ 0.01) by approximately 0.01 with each increase in added fat level. However, when 15 mg/kg narasin was fed, there were no differences (*P* ≥ 0.06) in G:F between pigs fed 0% and 1.3% added fat, or between pigs fed 2.6% and 4% added fat. Adding narasin at 15 mg/kg improved G:F by 3.18% (*P* < 0.01) with 0% added fat but provided no additional benefits (*P* = 1.00) when fed with 4% added fat. The additive benefits of feeding narasin diminished on G:F as fat level increased and it may not be beneficial to include both additional fat and narasin at the same time to growing-finishing pigs.

## Teaser

Narasin (Skycis) is an ionophore fed to growing/finishing pigs to improve growth rate and feed conversion. However, the benefits of narasin may diminish when fed in conjunction with diets containing higher fat (i.e., greater energy) levels. The objective of this trial was to evaluate the interactive effects of increasing energy by increasing corn oil (tested fat source) inclusion in the diets of finishing barrows and gilts fed with and without narasin.

## INTRODUCTION

Narasin (Skycis®, Elanco Animal Health, Greenfield, IN) is an ionophore used in the United States pork industry to increase rate of weight gain (ADG) of growing-finishing pigs when included in the complete feed at 15 mg/kg and to increase feed efficiency when fed at least 20 mg/kg for at least 4 wk before slaughter. Ionophores, like narasin, can improve performance by reducing gram-positive bacteria in the pig, which increases the gram-negative bacteria concentration and improves growth. As a result, a higher concentration of propionic acid is produced allowing for improved nutrient utilization (Kerr et al., 2017; Becker et al., 2024) which thereby improves growth performance.

It is also well established that adding dietary fat (energy) can improve ADG and feed efficiency of pigs (De la Llata et al., 2001; Stephenson et al., 2016; Marcal et al., 2019). De la Llata et al. (2001) reported ADG improved linearly (*P* < 0.01) due to fat addition over the total experiment. Pigs fed 4% added fat gained 5.7% more kg per day compared to pigs fed no added fat (De la Llata et al., 2001). De la Llata et al. (2001) also reported gain-to-feed ratio (G:F) increased linearly (*P* < 0.01) due to fat addition in all weight intervals and over the total experiment. Feed efficiency improved by 8.0% (*P* = 0.01) for pigs fed 4% added fat compared to pigs fed 0% added fat (De la Llata et al., 2001). In other scenarios, increasing metabolizable energy (ME) by 6% resulted in better performance of early finishing pigs (Stephenson et al., 2016). Diets with 4% added fat increased ADG (*P* = 0.05) by 3.8% and did not affect feed intake. This resulted in a 2.9% improvement in G:F (*P* < 0.01; Stephenson et al., 2016).

Even though narasin and added fat improve growth performance of growing-finishing pigs, the potential additive benefits are unknown. Both Rickard et al. (2017) and Arkfeld et al. (2015) fed 15 mg/kg narasin to grow/finish pigs in a multiphase finishing program. However, Rickard et al. (2017) fed 3,344 to 3,393 kcal/kg ME in the final two diet phases prior to marketing and reported a 1.1% improvement in ADG of pigs fed narasin (*P* < 0.05) compared to those not fed narasin. Arkfeld et al. (2015) fed a higher energy diet (3,390 to 3,400 kcal/kg ME) compared to Rickard et al. (2017) in the final two diet phases prior to marketing and reported that barrows fed narasin had a 2.0% greater overall ADG than barrows fed the control diet (*P* < 0.01), but ADG of gilts was not different (*P* = 0.69). The difference in results from Rickard et al. (2017) and Arkfeld et al. (2015) influenced the hypothesis of the current experiment that increased energy may dilute the response to narasin. Therefore, the objective was to evaluate the effects of feeding increasing levels of fat to growing-finishing pigs when fed with or without narasin.

## MATERIALS AND METHODS

All experimental procedures were reviewed and approved by the Carthage Veterinary Services IACUC committee (protocol #2024-23).

### Animals, Experimental Design, and Treatments

A total of 2,194 pigs (barrows and gilts; PIC 337 sire × PIC 1050 dam; Pig Improvement Company, Hendersonville, TN) with an initial body weight of 35.6 ± 3.6 kg were used in a 101-d feeding experiment at the end of the growing-finishing period. Dietary treatments were fed in a 4 × 2 factorial arrangement of treatments in a randomized complete block design. Factors included added fat (0.0%. 1.3%, 2.6%, and 4.0%) provided as corn oil and narasin (0.0 mg/kg or 15.0 mg/kg; Skycis, Elanco Animal Health, Greenfield, IN). A total of 88 mixed-sex pens divided across two rooms (40 pens in room 1 and 48 pens in room 2) were used with 11 replicates per treatment. Complete replicate blocks (a pen of pigs fed each treatment combination) were housed in the same area of the barn in the same room. Each treatment combination was represented in each replicate one time. Pen was the experimental unit for all measurements.

### Allotment to Study and Housing

Allotment to the study was performed when the pigs reached approximately 74 d of age (35.6 ± 3.16 kg BW). Rooms were stocked by arrival day at the farm and were 2 d apart. Each pen was stocked with a target of 25 pigs per pen and had an approximately equal number of barrows and gilts. Due to stocking density of the entire research site, one complete replicate block housed 26 pigs per pen. The research facility was a negative pressure, tunnel ventilated finishing barn with two rooms containing concrete slatted floors over a manure pit. Pens measured 5.39 m × 3.05 m with a 1.37 m^2^ single-sided dry box feeder and two nipple/cup drinkers. Each pen had moveable squeeze gates to standardize space per pig regardless of the number of pigs per pen. Squeeze gates were set to provide 0.65 m^2^ of space per pig when the pigs were allotted to treatment.

### Diets and Feeding

A four-phase dietary program was used during the study. Pigs were provided ad libitum access to feed and water for the duration of the trial. Diet changes were made at the end of each period (day 30, 54, and 80). All diets were formulated (Tables 1–4) to meet or exceed the current National Research Council’s (2012) guidelines. Diets were manufactured at a commercial feed mill in Carthage, IL. Samples of the diets were sent at the end of each period to a commercial laboratory (Midwest Laboratories, Omaha, NE) for compositional analyses. Diets were analyzed in duplicate for crude protein (AOAC 990.03, 2006), crude fat (AOAC 2003.05, 2007), neutral detergent fiber (Ankom Technology/AOAC 2001.11, 2005), ash (AOAC 942.05, 2012), calcium, phosphorous, sodium, iron, manganese, copper, and zinc (AOAC 985.01, 1996). Diet analyses are reported in Tables 5––8. Confirmation of narasin inclusion and exclusion in the complete feed was confirmed using a micro tracer rotary detector kit.

**Table 1.** Period 1^1^ diet formulation

	No Narasin (0.0 mg/kg)				Narasin (15.0 mg/kg)			
Ingredient, %	0.00% Fat	1.30% Fat	2.60% Fat	4.00% Fat	0.00% Fat	1.30% Fat	2.60% Fat	4.00% Fat
Corn	69.14	66.94	64.75	62.55	69.25	67.01	64.77	62.53
Soybean meal	17.95	18.83	19.72	20.60	17.90	18.80	19.70	20.60
DDGS (6% fat)	10.00	10.00	10.00	10.00	10.00	10.00	10.00	10.00
Fat (corn oil)	-	1.33	2.67	4.00	-	1.33	2.67	4.00
Calcium carbonate	1.03	1.02	1.02	1.02	1.03	1.03	1.02	1.02
Monocalcium phosphate 21%	0.21	0.20	0.19	0.19	0.21	0.20	0.19	0.19
Salt	0.56	0.56	0.56	0.56	0.56	0.56	0.56	0.56
L-Lysine HCl 78%	0.44	0.44	0.44	0.44	0.44	0.44	0.44	0.44
L-Threonine 99%	0.16	0.16	0.17	0.17	0.16	0.16	0.17	0.17
DL-Methionine 99%	0.18	0.16	0.14	0.12	0.10	0.11	0.11	0.12
L-Tryptophan 98.5%	0.04	0.04	0.04	0.04	0.04	0.04	0.04	0.04
L-Valine 98%	0.06	0.07	0.07	0.08	0.06	0.07	0.07	0.08
Vitamin/trace mineral premix	0.15	0.15	0.15	0.15	0.15	0.15	0.15	0.15
Skycis100	-	-	-	-	0.02	0.02	0.02	0.02
Microgrits blue—color	0.10	0.07	0.03	-	-	-	-	-
Microgrits red—color	-	0.03	0.07	0.10	-	-	-	-
Microgrits green—color	-	-	-	-	0.10	0.07	0.03	-
Microgrits orange—color	-	-	-	-	-	0.03	0.07	0.10

DDGS: Dried distillers grains with solubles.

^1^36 to 68 kg average body weight.

**Table 2.** Period 2^1^ diet formulation

	No Narasin (0.0 mg/kg)				Narasin (15.0 mg/kg)			
Ingredient, %	0.00% Fat	1.30% Fat	2.60% Fat	4.00% Fat	0.00% Fat	1.30% Fat	2.60% Fat	4.00% Fat
Corn	75.37	73.28	71.18	69.09	75.39	73.28	71.18	69.07
Soybean meal	12.00	12.77	13.53	14.30	12.00	12.77	13.53	14.30
DDGS (6% fat)	10.00	10.00	10.00	10.00	10.00	10.00	10.00	10.00
Fat (corn oil)	-	1.33	2.67	4.00	-	1.33	2.67	4.00
Calcium carbonate	0.19	0.18	0.18	0.17	0.19	0.18	0.18	0.17
Monocal phosphate 21%	0.97	0.96	0.96	0.96	0.97	0.96	0.96	0.96
Salt	0.55	0.55	0.55	0.55	0.55	0.55	0.55	0.55
L-Lysine HCl 78%	0.40	0.40	0.40	0.40	0.40	0.40	0.40	0.40
L-Threonine 99%	0.13	0.13	0.13	0.14	0.13	0.13	0.13	0.14
DL-Methionine 99%	0.09	0.08	0.08	0.07	0.06	0.06	0.07	0.07
L-Tryptophan 98.5%	0.04	0.04	0.04	0.04	0.04	0.04	0.04	0.04
L-Valine 98%	0.04	0.04	0.04	0.05	0.04	0.04	0.04	0.05
Vitamin/trace mineral premix	0.15	0.15	0.15	0.15	0.15	0.15	0.15	0.15
Skycis100	-	-	-	-	0.02	0.02	0.02	0.02
Microgrits blue—color	0.10	0.07	0.03	0.00	-	-	-	-
Microgrits red—color	-	0.03	0.07	0.10	-	-	-	-
Microgrits green—color	-	-	-	-	0.10	0.07	0.03	-
Microgrits orange—color	-	-	-	-	-	0.03	0.07	0.10

DDGS: Dried distillers grains with solubles.

^1^68 to 95 kg average body weight.

**Table 3.** Period 3^1^ diet formulation

	No Narasin (0.0 mg/kg)				Narasin (15.0 mg/kg)			
Ingredient, %	0.00% Fat	1.30% Fat	2.60% Fat	4.00% Fat	0.00% Fat	1.30% Fat	2.60% Fat	4.00% Fat
Corn	77.87	75.92	73.97	72.02	77.84	75.89	73.95	72.00
Soybean meal	9.90	10.50	11.10	11.70	9.90	10.50	11.10	11.70
DDGS (6% fat)	10.00	10.00	10.00	10.00	10.00	10.00	10.00	10.00
Fat (corn oil)	-	1.33	2.67	4.00	-	1.33	2.67	4.00
Calcium carbonate	0.05	0.05	0.05	0.05	0.05	0.05	0.05	0.05
Monocal phosphate 21%	0.91	0.91	0.90	0.90	0.91	0.90	0.90	0.90
Salt	0.55	0.55	0.55	0.55	0.55	0.55	0.55	0.55
L-Lysine HCl 78%	0.34	0.34	0.35	0.35	0.34	0.34	0.35	0.35
L-Threonine 99%	0.09	0.10	0.10	0.11	0.09	0.10	0.10	0.11
DL-Methionine 99%	0.01	0.02	0.03	0.04	0.03	0.03	0.03	0.04
L-Tryptophan 98.5%	0.03	0.03	0.03	0.03	0.03	0.03	0.03	0.03
L-Valine 98%	-	0.01	0.01	0.02	-	0.01	0.01	0.02
Vitamin/trace mineral premix	0.15	0.15	0.15	0.15	0.15	0.15	0.15	0.15
Skycis100	-	-	-	-	0.02	0.02	0.02	0.02
Microgrits blue—color	0.10	0.07	0.03	-	-	-	-	-
Microgrits red—color	-	0.03	0.07	0.10	-	-	-	-
Microgrits green—color	-	-	-	-	0.10	0.07	0.03	-
Microgrits orange—color	-	-	-	-	-	0.03	0.07	0.10

DDGS: Dried distillers grains with solubles.

^1^95 to 124 kg average body weight.

**Table 4.** Period 4^1^ diet formulation

	No Narasin (0.0 mg/kg)				Narasin (15.0 mg/kg)			
Ingredient, %	0.00% Fat	1.30% Fat	2.60% Fat	4.00% Fat	0.00% Fat	1.30% Fat	2.60% Fat	4.00% Fat
Corn	78.35	76.32	74.30	72.27	78.35	76.32	74.29	72.26
Soybean meal	9.50	10.20	10.90	11.60	9.50	10.20	10.90	11.60
DDGS (6% fat)	10.00	10.00	10.00	10.00	10.00	10.00	10.00	10.00
Fat (corn oil)	-	1.33	2.67	4.00	-	1.33	2.67	4.00
Calcium carbonate	0.95	0.93	0.92	0.91	0.93	0.92	0.92	0.91
Salt	0.55	0.55	0.55	0.55	0.55	0.55	0.55	0.55
L-Lysine HCl 78%	0.30	0.30	0.30	0.30	0.30	0.30	0.30	0.30
L-Threonine 99%	0.08	0.08	0.08	0.09	0.08	0.08	0.08	0.09
DL-Methionine 99%	-	-	0.01	0.01	-	-	0.01	0.01
L-Tryptophan 98.5%	0.03	0.02	0.02	0.02	0.03	0.02	0.02	0.02
Vitamin/trace mineral premix	0.15	0.15	0.15	0.15	0.15	0.15	0.15	0.15
Skycis100	-	-	-	-	0.02	0.02	0.02	0.02
Microgrits blue—color	0.10	0.07	0.03	-	-	-	-	-
Microgrits red—color	-	0.03	0.07	0.10	-	-	-	-
Microgrits green—color	-	-	-	-	0.10	0.07	0.03	-
Microgrits orange—color	-	-	-	-	-	0.03	0.07	0.10

DDGS: Dried distillers grains with solubles.

^1^124 to 138 kg average body weight.

**Table 5.** Period 1^1^ nutrient composition of experimental diets (as-fed basis)

	No Narasin (0.0 mg/kg)								Narasin (15.0 mg/kg)							
	0.00% Fat		1.30% Fat		2.60% Fat		4.00% Fat		0.00% Fat		1.30% Fat		2.60% Fat		4.00% Fat	
Composition^2^^,^^3^	Calc.	Analy.	Calc.	Analy.	Calc.	Analy.	Calc.	Analy.	Calc.	Analy.	Calc.	Analy.	Calc.	Analy.	Calc.	Analy.
Mod ME, kcal/kg	3197	-	3267	-	3336	-	3406	-	3195	-	3265	-	3335	-	3405	-
Mod NE, kcal/kg	2515	-	2579	-	2646	-	2709	-	2513	-	2579	-	2643	-	2709	-
Dry Matter	86.55	85.21	86.77	85.69	86.98	85.67	87.20	85.75	86.53	85.64	86.75	86.21	86.97	86.43	87.19	86.16
Crude Protein, %	16.93	16.90	17.18	18.20	17.43	17.30	17.68	18.30	16.87	17.70	17.14	18.40	17.41	18.20	17.68	17.30
Crude Fat, %	3.41	3.54	4.61	4.09	5.81	5.46	7.01	6.16	3.42	3.08	4.62	4.12	5.81	5.32	7.01	5.86
Ash, %	4.13	3.86	4.15	3.90	4.17	3.95	4.19	3.87	4.14	3.98	4.16	4.32	4.17	4.26	4.19	4.09
NDF, %	10.14	9.70	10.03	10.10	9.91	8.90	9.80	8.70	10.15	10.60	10.03	9.80	9.92	9.40	9.80	9.00
Analyzed Ca, %	0.53	0.48	0.53	0.43	0.53	0.56	0.53	0.53	0.53	0.60	0.53	0.59	0.53	0.47	0.53	0.50
Phosphorus, %	0.42	0.45	0.42	0.46	0.42	0.43	0.42	0.44	0.42	0.46	0.42	0.46	0.42	0.44	0.42	0.45
Ca:P	1.25	1.07	1.25	0.93	1.25	1.30	1.25	1.20	1.26	1.30	1.26	1.28	1.25	1.07	1.25	1.11
STTD Phosphorus, %	0.34	-	0.34	-	0.34	-	0.34	-	0.34	-	0.34	-	0.34	-	0.34	-
Phytase, FTU/kg	749.56	-	749.56	-	749.56	-	749.56	-	749.56	-	749.56	-	749.56	-	749.56	-
SID Lysine, %	0.96	-	0.98	-	1.00	-	1.02	-	0.96	-	0.98	-	1.00	-	1.02	-
SID Lys:Cal ME ratio	3.00	-	3.00	-	3.00	-	3.00	-	3.00	-	3.00	-	3.00	-	3.00	-
SID Lys:Cal NE ratio	3.83	-	3.81	-	3.79	-	3.77	-	3.82	-	3.80	-	3.79	-	3.77	-
SID AA:SID Lys (calculated ratio)																
Ile:Lys	0.56	-	0.56	-	0.56	-	0.56	-	0.56	-	0.56	-	0.56	-	0.56	-
Leu:Lys	1.38	-	1.36	-	1.35	-	1.33	-	1.38	-	1.36	-	1.35	-	1.33	-
Met + Cys:Lys	0.66	-	0.63	-	0.61	-	0.58	-	0.58	-	0.58	-	0.58	-	0.58	-
Thr:Lys	0.65	-	0.65	-	0.65	-	0.65	-	0.65	-	0.65	-	0.65	-	0.65	-
Trp:Lys	0.19	-	0.19	-	0.19	-	0.19	-	0.19	-	0.19	-	0.19	-	0.19	-
Val:Lys	0.70	-	0.70	-	0.70	-	0.70	-	0.70	-	0.70	-	0.70	-	0.70	-

AA: amino acid, Mod ME: modified metabolizable energy, NE: net energy, SID: standardized ileal digestible, STTD: standardized total tract digestible.

^1^36 to 68 kg average body weight.

^2^Diets were in meal form and manufactured at the NSI feed mill (Carthage, IL)^.^

^3^Analyses were carried out by Midwest Labs (Omaha, NE) using wet chemistry.

**Table 6.** Period 2^1^ nutrient composition of experimental diets (as-fed basis)

	No Narasin (0.0 mg/kg)								Narasin (15.0 mg/kg)							
	0.00% Fat		1.30% Fat		2.60% Fat		4.00% Fat		0.00% Fat		1.30% Fat		2.60% Fat		4.00% Fat	
Composition^1^^,^^2^	Calc.	Analy.	Calc.	Analy.	Calc.	Analy.	Calc.	Analy.	Calc.	Analy.	Calc.	Analy.	Calc.	Analy.	Calc.	Analy.
Mod ME, kcal/kg	3201	-	3271	-	3341	-	3411	-	3200	-	3270	-	3341	-	3411	-
Mod NE, kcal/kg	2522	-	2588	-	2652	-	2718	-	2522	-	2588	-	2652	-	2718	-
Dry Matter	86.36	86.37	86.57	86.47	86.79	86.64	87.00	86.94	86.34	86.49	86.56	87.03	86.77	86.78	86.99	86.80
Crude Protein, %	14.49	15.20	14.70	14.10	14.90	15.10	15.11	14.40	14.47	14.30	14.68	14.00	14.90	14.40	15.11	14.20
Crude Fat, %	3.56	3.42	4.76	4.36	5.96	5.62	7.16	6.57	3.56	3.28	4.76	4.35	5.96	5.50	7.16	6.45
Ash, %	3.76	3.50	3.77	3.32	3.79	3.50	3.80	3.33	3.76	3.57	3.77	3.55	3.79	3.42	3.80	3.66
NDF, %	10.21	9.00	10.09	9.20	9.98	7.90	9.86	7.70	10.21	7.70	10.09	7.80	9.98	8.10	9.86	8.20
Analyzed Ca, %	0.49	0.55	0.49	0.37	0.49	0.57	0.49	0.46	0.49	0.59	0.49	0.48	0.49	0.51	0.49	0.59
Phosphorus, %	0.39	0.43	0.39	0.41	0.39	0.42	0.39	0.41	0.39	0.40	0.39	0.40	0.39	0.42	0.39	0.39
Ca:P	1.25	1.28	1.25	0.90	1.25	1.36	1.25	1.12	1.25	1.48	1.25	1.20	1.25	1.21	1.25	1.51
STTD Phosphorus, %	0.32	-	0.32	-	0.32	-	0.32	-	0.32	-	0.32	-	0.32	-	0.32	-
Phytase, FTU/kg	749.56	-	749.56	-	749.56	-	749.56	-	749.56	-	749.56	-	749.56	-	749.56	-
SID Lys:Cal ME ratio	2.47	-	2.47	-	2.47	-	2.47	-	2.47	-	2.47	-	2.47	-	2.47	-
SID Lys:Cal NE ratio	3.14	-	3.13	-	3.11	-	3.10	-	3.14	-	3.13	-	3.11	-	3.10	-
SID AA:SID Lys (calculated ratio)																
Ile:Lys	0.56	-	0.56	-	0.56	-	0.56	-	0.56	-	0.56	-	0.56	-	0.56	-
Leu:Lys	1.51	-	1.49	-	1.46	-	1.44	-	1.51	-	1.49	-	1.46	-	1.44	-
Met + Cys:Lys	0.63	-	0.61	-	0.60	-	0.58	-	0.59	-	0.59	-	0.58	-	0.58	-
Thr:Lys	0.65	-	0.65	-	0.65	-	0.65	-	0.65	-	0.65	-	0.65	-	0.65	-
Trp:Lys	0.19	-	0.19	-	0.19	-	0.19	-	0.19	-	0.19	-	0.19	-	0.19	-
Val:Lys	0.70	-	0.70	-	0.70	-	0.70	-	0.70	-	0.70	-	0.70	-	0.70	-

AA: amino acid, Mod ME: modified metabolizable energy, NE: net energy, SID: standardized ileal digestible, STTD: standardized total tract digestible.

^1^68 to 95 kg average body weight.

^2^Diets were in meal form and manufactured at the NSI feed mill (Carthage, IL)^.^

^3^Analyses were carried out by Midwest Labs (Omaha, NE) using wet chemistry.

**Table 7.** Period 3^1^ nutrient composition of experimental diets (as-fed basis)

	No Narasin (0.0 mg/kg)								Narasin (15.0 mg/kg)							
	0.00% Fat		1.30% Fat		2.60% Fat		4.00% Fat		0.00% Fat		1.30% Fat		2.60% Fat		4.00% Fat	
Composition^2^^,^^3^	Calc.	Analy.	Calc.	Analy.	Calc.	Analy.	Calc.	Analy.	Calc.	Analy.	Calc.	Analy.	Calc.	Analy.	Calc.	Analy.
Mod ME, kcal/kg	3206	-	3276	-	3346	-	3416	-	3206	-	3276	-	3346	-	3416	-
Mod NE, kcal/kg	2529	-	2593	-	2659	-	2723	-	2529	-	2593	-	2659	-	2723	-
Dry Matter	86.25	85.95	86.46	86.35	86.68	86.66	86.89	86.93	86.24	86.65	86.45	86.70	86.67	86.51	86.88	86.40
Crude Protein, %	13.54	13.70	13.69	13.40	13.85	13.60	14.00	14.10	13.55	14.30	13.70	12.90	13.85	13.90	14.00	14.00
Crude Fat, %	3.62	3.34	4.82	4.62	6.03	5.94	7.23	6.75	3.62	3.37	4.82	4.70	6.03	5.59	7.23	7.02
Ash, %	3.47	3.23	3.48	3.14	3.49	3.36	3.50	3.28	3.47	3.64	3.48	3.65	3.49	3.35	3.50	3.40
NDF, %	10.26	9.20	10.14	7.80	10.03	8.90	9.91	8.70	10.25	8.80	10.14	9.00	10.02	9.40	9.91	8.40
Analyzed Ca, %	0.45	0.37	0.45	0.47	0.44	0.49	0.44	0.47	0.45	0.50	0.45	0.51	0.44	0.38	0.44	0.45
Phosphorus, %	0.36	0.39	0.36	0.37	0.36	0.36	0.36	0.35	0.36	0.36	0.36	0.34	0.36	0.37	0.36	0.37
Ca:P	1.25	0.95	1.25	1.27	1.25	1.36	1.25	1.34	1.25	1.39	1.25	1.50	1.25	1.03	1.25	1.22
STTD Phosphorus, %	0.29	-	0.29	-	0.29	-	0.29	-	0.29	-	0.29	-	0.29	-	0.29	-
Phytase, FTU/kg	749.56	-	749.56	-	749.56	-	749.56	-	749.56	-	749.56	-	749.56	-	749.56	-
SID Lysine, %	0.70	-	0.71	-	0.73	-	0.74	-	0.70	-	0.71	-	0.73	-	0.74	-
SID Lys:Cal ME ratio	2.18	-	2.18	-	2.17	-	2.17	-	2.18	-	2.18	-	2.17	-	2.17	-
SID Lys:Cal NE ratio	2.77	-	2.76	-	2.74	-	2.73	-	2.77	-	2.76	-	2.74	-	2.73	-
SID AA:SID Lys (calculated ratio)																
Ile:Lys	0.59	-	0.59	-	0.58	-	0.58	-	0.59	-	0.59	-	0.58	-	0.58	-
Leu:Lys	1.64	-	1.61	-	1.59	-	1.56	-	1.64	-	1.61	-	1.59	-	1.56	-
Met + Cys:Lys	0.58	-	0.58	-	0.58	-	0.58	-	0.61	-	0.60	-	0.59	-	0.58	-
Thr:Lys	0.65	-	0.65	-	0.65	-	0.65	-	0.65	-	0.65	-	0.65	-	0.65	-
Trp:Lys	0.19	-	0.19	-	0.19	-	0.19	-	0.19	-	0.19	-	0.19	-	0.19	-
Val:Lys	0.70	-	0.70	-	0.70	-	0.70	-	0.70	-	0.70	-	0.70	-	0.70	-

AA: amino acid, Mod ME: modified metabolizable energy, NE: net energy, SID: standardized ileal digestible, STTD: standardized total tract digestible.

^1^95 to 124 kg average body weight.

^2^Diets were in meal form and manufactured at the NSI feed mill (Carthage, IL)^.^

^3^Analyses were carried out by Midwest Labs (Omaha, NE) using wet chemistry.

**Table 8.** Period 4^1^ nutrient composition of experimental diets (as-fed basis)

	No Narasin (0.0 mg/kg)								Narasin (15.0 mg/kg)							
	0.00% Fat		1.30% Fat		2.60% Fat		4.00% Fat		0.00% Fat		1.30% Fat		2.60% Fat		4.00% Fat	
Composition^2^^,^^3^	Calc.	Analy.	Calc.	Analy.	Calc.	Analy.	Calc.	Analy.	Calc.	Analy.	Calc.	Analy.	Calc.	Analy.	Calc.	Analy.
Mod ME, kcal/kg	3205	-	3275	-	3346	-	3416	-	3205	-	3275	-	3346	-	3416	-
Mod NE, kcal/kg	2529	-	2593	-	2659	-	2723	-	2529	-	2593	-	2659	-	2723	-
Dry Matter	86.23	85.72	86.44	85.99	86.66	86.06	86.87	86.22	86.21	86.09	86.42	86.26	86.64	86.24	86.85	86.66
Crude Protein, %	13.33	13.60	13.51	12.40	13.70	12.50	13.88	14.50	13.33	12.50	13.51	12.90	13.69	13.80	13.87	13.50
Crude Fat, %	3.63	2.89	4.83	4.34	6.04	5.56	7.24	6.50	3.63	3.22	4.83	4.48	6.04	5.72	7.24	6.74
Ash, %	3.44	3.12	3.45	3.14	3.45	3.28	3.46	3.36	3.43	2.95	3.44	3.14	3.45	2.93	3.46	3.23
NDF, %	10.27	8.20	10.15	8.10	10.04	8.10	9.92	7.80	10.27	8.80	10.15	6.80	10.04	7.20	9.92	7.00
Analyzed Ca, %	0.45	0.53	0.45	0.49	0.44	0.42	0.44	0.40	0.45	0.46	0.45	0.52	0.44	0.42	0.44	0.54
Phosphorus, %	0.34	0.34	0.34	0.33	0.34	0.33	0.34	0.35	0.34	0.35	0.34	0.34	0.34	0.35	0.34	0.33
Ca:P	1.31	1.56	1.30	1.48	1.29	1.27	1.28	1.14	1.29	1.31	1.29	1.53	1.28	1.20	1.28	1.64
STTD Phosphorus, %	0.28	-	0.28	-	0.28	-	0.28	-	0.28	-	0.28	-	0.28	-	0.28	-
Phytase, FTU/kg	749.56	-	749.56	-	749.56	-	749.56	-	749.56	-	749.56	-	749.56	-	749.56	-
SID Lys:Cal ME ratio	2.05	-	2.05	-	2.05	-	2.05	-	2.05	-	2.05	-	2.05	-	2.05	-
SID Lys:Cal NE ratio	2.60	-	2.59	-	2.59	-	2.58	-	2.60	-	2.59	-	2.59	-	2.58	-
SID AA:SID Lys (calculated ratio)																
Ile:Lys	0.61	-	0.61	-	0.61	-	0.61	-	0.61	-	0.61	-	0.61	-	0.61	-
Leu:Lys	1.73	-	1.70	-	1.68	-	1.65	-	1.73	-	1.70	-	1.68	-	1.65	-
Met + Cys:Lys	0.59	-	0.59	-	0.58	-	0.58	-	0.59	-	0.59	-	0.58	-	0.58	-
Thr:Lys	0.66	-	0.66	-	0.66	-	0.66	-	0.66	-	0.66	-	0.66	-	0.66	-
Trp:Lys	0.19	-	0.19	-	0.19	-	0.19	-	0.19	-	0.19	-	0.19	-	0.19	-
Val:Lys	0.73	-	0.73	-	0.72	-	0.72	-	0.73	-	0.73	-	0.72	-	0.72	-

AA: amino acid, Mod ME: modified metabolizable energy, NE: net energy, SID: standardized ileal digestible, STTD: standardized total tract digestible.

^1^124 to 138 kg average body weight.

^2^Diets were in meal form and manufactured at the NSI feed mill (Carthage, IL)^.^

^3^Analyses were carried out by Midwest Labs (Omaha, NE) using wet chemistry.

### Data Collection

Body weight and feed intake were recorded on a pen basis and summarized at d 0 (start of experimental feeding period), 30, 54, and 80 to calculate ADG and average daily feed intake (ADFI). Gain to feed ratio was calculated using these data. The percentage of pigs removed was also calculated for each phase and cumulatively. The date, weight, and reason for mortality or morbidity were recorded for all pigs removed from the study.

### Marketing Strategy

Pigs were sent for slaughter (d 80, d 87, d 94, and d 101) according to the following marketing strategy: 1) on d 80 of the study, the heaviest 20% of pigs from each pen was sent for slaughter, 2) on d 87 of the study, the next heaviest 25% of each pen was sent for slaughter, 3) on d 94 of the study, the next heaviest 25% of each pen was sent for slaughter, and 4) on d 101 of the study, the remaining 30% of each pen was sent for slaughter. Pen inventory within each replicate block was standardized at each marketing event. Pigs within each pen were visually appraised for weight to be selected for slaughter by the production site’s normal marketing personnel. On each day that pigs were sent for slaughter, pigs that were selected for slaughter were weighed as a group, loaded on a conventional semi-trailer, and shipped to a commercial slaughter facility.

### Statistical Analysis

Analyses were conducted using the lme4 package (v1.1-36; Bates et al., 2015) in R version 4.4.2. Pigs were allocated to pens so the starting variation and weights were approximately equal across pens within each replicate block. The replicate block was included in the models as a random intercept. Pen served as the experimental unit for all response parameters. Treatments were assigned to pens by replicate block in a randomized complete block design. Data were analyzed as a 4 × 2 factorial arrangement of treatments coded by added fat level (0.0%, 1.3%, 2.6%, vs 4.0%) and narasin level (0.0 mg/kg vs 15.0 mg/kg) and their interactions as fixed factors. The replicate block was included as a random variable in the model. Estimated marginal means and pairwise comparisons from the models were estimated with the emmeans package (v1.10.3; Lenth, 2016) with a Tukey-Kramer adjustment for multiple comparisons. Uncertainty of the estimated marginal means was expressed as the maximum standard error among each of the 4 × 2 treatment combinations. Estimated marginal mean differences for treatments were considered statistically significant at *P* ≤ 0.05.

## RESULTS

### Overall Growth Performance

**Figure 1.**
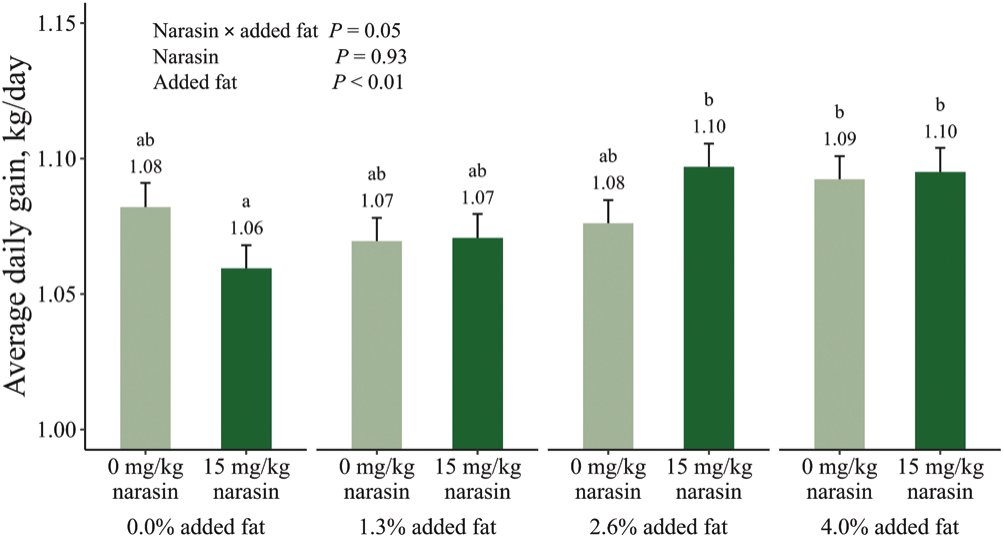
Interactive estimated marginal means for average daily gain of growing-finishing pigs fed increasing levels of added fat (corn oil) with 15 mg/kg narasin (trade name Skycis, Elanco Animal Health, Greenfield, In) or without narasin during a 113 d feeding period. Treatment means that do not share a superscript differ (*P* ≤ 0.05).

**Figure 2.**
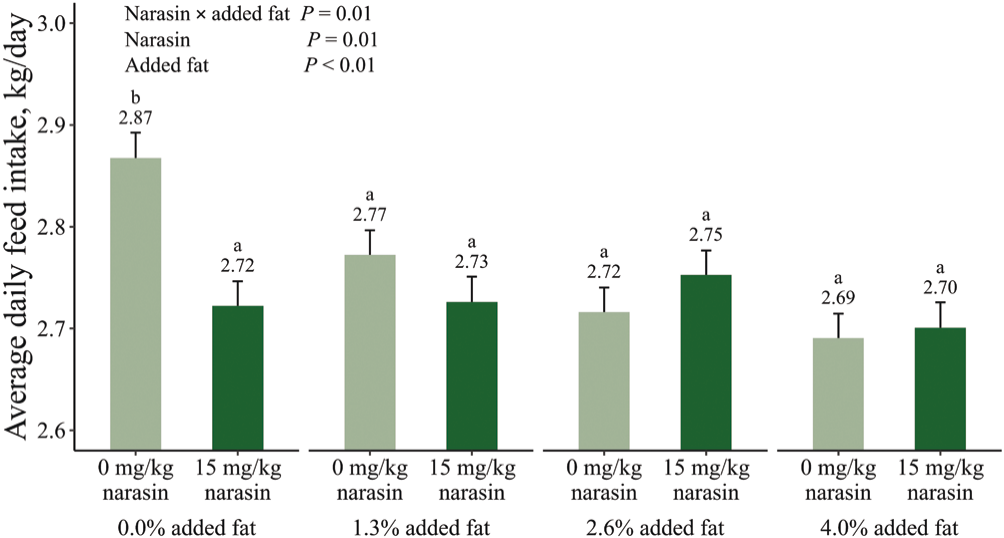
Interactive estimated marginal means for average daily feed intake of growing-finishing pigs fed increasing levels of added fat (corn oil) with 15 mg/kg narasin (trade name Skycis, Elanco Animal Health, Greenfield, In) or without narasin during a 113 d feeding period. Treatment means that do not share a superscript differ (*P* ≤ 0.05).

**Figure 3.**
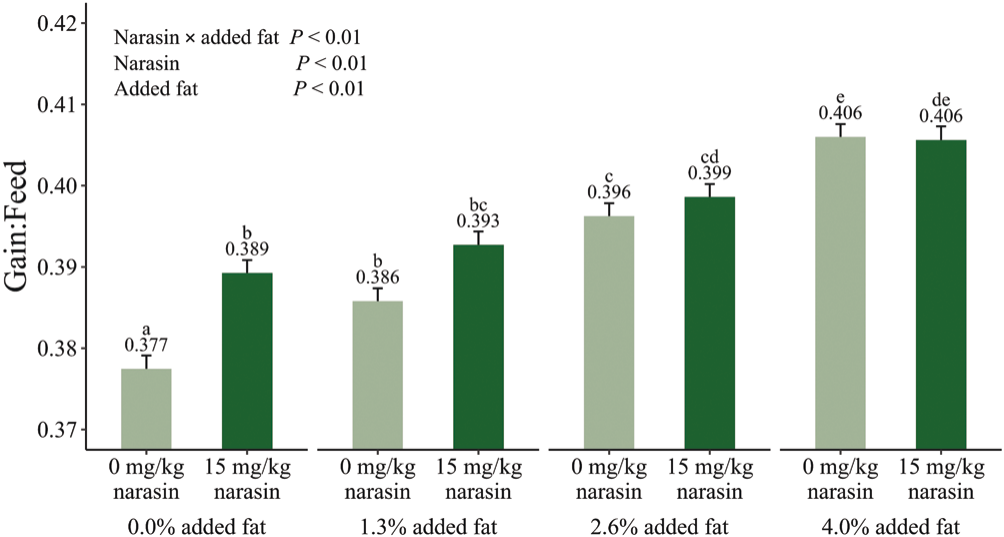
Interactive least squares means for gain:feed of growing-finishing pigs fed increasing levels of added fat (corn oil) with 15 mg/kg narasin (trade name Skycis, Elanco Animal Health, Greenfield, In) or without narasin during a 113 d feeding period. Treatment means that do not share a superscript differ (*P* ≤ 0.05).

The interactions between added fat and narasin on cumulative growth performance traits are shown in Figures 1–3. Main effects of added fat and narasin are provided in Supplemental Fig 1–3. There was an interaction (*P* = 0.05) between added fat and narasin on cumulative ADG (Figure 1). Pigs that were fed 0% added fat and 15 mg/kg narasin gained 0.03 to 0.04 fewer kg per day (*P* ≤ 0.05) compared to pigs fed 2.6% added fat and 15 mg/kg narasin and pigs fed 4% added fat with narasin or no narasin. There was a significant linear effect of fat on ADG when 15 mg/kg narasin was included in the diets (*P *< 0.01) which was not present without narasin (*P *= 0.27). There was also an interaction (*P* < 0.01) between added fat and narasin for cumulative ADFI (Figure 2). Pigs fed 0% added fat and no narasin ate at least 0.10 more kg per day (*P* ≤ 0.03) compared to all other treatments. There was a significant linear effect of fat on ADFI when narasin was not included in the diets (*P *< 0.01) which was not present when 15 mg/kg narasin was included (*P *= 0.67). There was an interaction (*P* < 0.01) between added fat and narasin for cumulative G:F (Figure 3). Pigs fed 0% added fat and no narasin had the lowest (*P* ≤ 0.01) G:F by at least 0.009 compared to all other treatments. The G:F of pigs fed 0 mg/kg narasin increased (*P* ≤ 0.01) by approximately 0.01 with each increase in added fat level. However, when 15 mg/kg narasin was fed, there were no differences (*P* ≥ 0.06) in G:F between pigs fed 0% and 1.3% added fat, or between pigs fed 2.6% and 4% added fat. Adding narasin at 15 mg/kg improved G:F by 3.18% (*P* < 0.01) with 0% added fat but provided no additional benefits (*P* = 1.00) when fed with 4% added fat. There was a significant linear effect of fat on G:F regardless of whether narasin was included in the diets (*P *< 0.01). The slope when narasin was not included in the diets was 0.007 units G:F per increase in percent fat compared to 0.004 units G:F per increase in percent fat when 15 mg/kg narasin was included, resulting in a difference of 0.003 units G:F per increase in percent fat (*P* < 0.01). This difference is likely due to improved G:F in pigs fed 15 mg/kg narasin at decreased added fat inclusions.

### Period 1 Growth Performance (d 0 to 30)

The interactive means for growth performance from each period and marketing are displayed in Table 9. From d 0 to 30, there was an interaction (*P* = 0.05) for ADFI, however pairwise comparisons were insignificant (P ≥ 0.19). There were no interactions (*P* ≥ 0.19) for average pig weight, ADG, or G:F. There was a main effect (*P* < 0.01) of fat inclusion on average pig body weight on d 30. Pigs fed 4% added fat weighed at least 0.93 kg more (*P* ≤ 0.02) compared to pigs fed 1.3% or 0% added fat. Similarly, ADG was increased (*P* < 0.01) by at least 0.04 kg for pigs fed 4% added fat compared to 1.3% or 0%. Pigs fed 4% added fat also had greater G:F (*P* < 0.01) by at least 0.02 compared to pigs fed 1.3% or 0% added fat.

**Table 9.** Interactive least squares means for growing-finishing performance of pigs fed increasing levels of fat with or without narasin

	No Narasin (0 mg/kg)				Narasin (15.0 mg/kg)					^1^ *P*-value		
Item	Fat 0.00%	Fat 1.30%	Fat 2.60%	Fat 4.00%	Fat 0.00%	Fat 1.30%	Fat 2.60%	Fat 4.00%	SEM	Narasin × Fat	Narasin	Fat
Start Weight^2^	35.62	35.61	35.52	35.56	35.55	35.59	35.77	35.54	0.44	0.53	0.69	0.88
Period 1 (31 d)												
End weight, kg	67.27	67.09	67.75	68.38	66.67	67.69	68.4	68.35	0.58	0.19	0.50	<0.01
ADG^3^, kg	1.01	1.01	1.03	1.05	1.00	1.03	1.06	1.06	0.01	0.30	0.24	<0.01
ADFI^3^, kg	2.17	2.13	2.12	2.10	2.12	2.15	2.17	2.14	0.03	0.05	0.39	0.45
G:F^3^	0.467	0.474	0.487	0.501	0.473	0.478	0.488	0.497	0.006	0.63	0.58	<0.01
Removal, (%, rem/start)	0.72(2/276)	0.73(2/273)	0.74(2/272)	0.73(2/274)	0.36(1/274)	1.82(5/275)	0.36(1/275)	0.73(2/275)				
Period 2 (24 d)												
End weight, kg	94.94	94.9	94.87	95.89	93.97	95.02	96.37	96.12	0.75	0.07	0.50	0.01
ADG^3^, kg	1.15	1.14	1.13	1.15	1.13	1.14	1.17	1.16	0.02	0.16	0.33	0.74
ADFI^3^, kg	2.97^b^	2.85^a^	2.78^a^	2.77^a^	2.81^a^	2.82^a^	2.84^a^	2.75^a^	0.04	<0.01	0.06	<0.01
G:F^3^	0.390	0.400	0.409	0.415	0.404	0.406	0.412	0.424	0.012	0.59	0.01	<0.01
Removal, (%, rem/start)	2.19(6/274)	1.48(4/271)	0.37(1/270)	0.37(1/272)	0.73(2/273)	0.37(1/270)	0.73(2/274)	0.37(1/273)				
Period 3 (26 d)												
End weight, kg	123.48^ab^	123.26^ab^	122.77^ab^	124.54^ab^	122.29^a^	122.98^ab^	125.09^b^	124.74^ab^	0.67	0.04	0.54	0.02
ADG^3^, kg	1.09	1.10	1.08	1.11	1.08	1.08	1.10	1.11	0.01	0.55	0.91	0.46
ADFI^3^, kg	3.31^c^	3.24^bc^	3.13^ab^	3.11^ab^	3.14^ab^	3.12^ab^	3.15^ab^	3.10^a^	0.08	0.02	<0.01	<0.01
G:F^3^	0.332	0.340	0.346	0.358	0.346	0.346	0.351	0.359	0.006	0.14	<0.01	<0.01
Removal, (%, rem/start)	1.87(5/268)	0.37(1/267)	0.00(0/269)	0.74(2/271)	0.74(2/271)	0.37(1/269)	0.74(2/272)	0.37(1/272)				
Marketing^4^ (21 d)												
End weight, kg	136.15	135.56	135.44	136.95	134.26	135.11	137.47	136.97	0.65	0.08	0.89	0.06
ADG^3^, kg	1.05	1.03	1.07	1.05	1.01	1.02	1.05	1.05	0.02	0.78	0.23	0.30
ADFI^3^, kg	3.52^b^	3.38^ab^	3.32^a^	3.22^a^	3.28^a^	3.26^a^	3.31^a^	3.29^a^	0.04	<0.01	0.01	0.01
G:F^3^^,^^5^	0.299	0.309	0.305	0.314	0.324	0.317	0.325	0.319	0.003	0.06	0.59	<0.01

^1^Treatments within a row that do not share a superscript differ (*P* < 0.05).

^2^Weights do not incorporate pig mortality between periods and represent surviving pigs only at each weigh period.

^3^ADG, ADFI, and G:F were corrected for mortality by adding weights of all pigs in the period and dividing by the cumulative days each pig was in the pen from the start of the weight period.

^4^Pigs were marketed in a series of 4 market cuts occurring approximately every 7 to 8 d.

^5^Replicate random effect was removed to facilitate model convergence.

### Period 2 Growth Performance (d 31 to 55)

There was a main effect (*P* = 0.01) of fat on weight at d 55. Pigs fed 4% added fat weighed 1.56 kg more (*P* < 0.01) compared to pigs fed 0% added fat. Pigs fed 4% added fat also ate at least 0.08 kg less (*P* ≤ 0.04) per day compared to pigs fed 1.3% or 0% added fat. There was also an interaction (*P* < 0.01) between narasin and fat level on feed intake. Pigs fed no narasin and 0% added fat ate at least 0.12 kg more (*P* ≤ 0.03) per day compared to all other treatments. Gain-to-feed ratio was increased by 0.007 for pigs fed 15.0 mg/kg narasin compared to no narasin. Gain-to-feed ratio was also increased (*P* < 0.01) by at least 0.017 for pigs fed 4% added fat compared to 1.3% and 0% added fat.

### Period 3 Growth Performance (d 56 to 80)

There was an interaction between narasin and added fat (*P* = 0.04) on weight at d 82. Pigs fed 15.0 mg/kg narasin weighed 2.80 kg less (*P* = 0.04) when fed 2.6% added fat compared to 0% added fat. There was also an interaction (*P* = 0.02) on ADFI. Pigs fed no narasin and no added fat increased (*P* ≤ 0.03) their feed intake by at least 0.16 kg per day compared to pigs fed all treatments containing narasin and pigs fed no narasin with 2.6% or 4% added fat. There were main effects of narasin (*P* < 0.01) and added fat (*P* < 0.01) on G:F in period 3. Pigs fed narasin increased (*P* < 0.01) G:F by 0.006 compared to pigs not fed narasin. Pigs fed 2.6% added fat increased (*P* = 0.01) G:F by 0.009 compared to pigs fed 0% added fat. Pigs fed 4% added fat increased (*P* < 0.01) G:F by at least 0.01 compared to pigs fed the other 3 fat levels.

### Marketing (d 81 to 102)

There was an interaction (*P* < 0.01) on ADFI during marketing. Pigs fed no narasin and no added fat ate at least 0.2 kg less (*P* ≤ 0.01) per day compared to pigs fed all treatments containing narasin and pigs fed no narasin with 2.6% or 4% added fat. There was a main effect (*P* < 0.01) of fat inclusion on G:F during the marketing phase. Pigs fed 2.6% or 4% added fat increased (*P* ≤ 0.05) G:F by at least 0.01 compared to pigs fed 1.3% or 0% added fat.

## DISCUSSION

Narasin is an ionophore that alters microbial fermentation in the cecum and colon of swine. The shift in microbial population allows for better energy utilization by increasing the relative level of propionate compared to acetate and butyrate through the selective inhibition of gram-positive bacteria, resulting in more efficient fermentation (Wuethrich et al., 1998). The results of this study demonstrated that the additive effects of narasin diminished when dietary fat was included in the diet, which is consistent with the initial hypothesis. At the time this study was conducted, soybean oil was trading for around $0.99 per kg (Business Insider, 2024a) and lean hogs were trading for around $2.05 per kg at the time of marketing (Business Insider, 2024b). The addition of 4% fat is less profitable in this scenario as the producer pays an increased daily cost of $0.08 for the added fat but only returns an added $0.02 in daily gain leading to a daily decrease of $0.05. In this case, narasin may be used to better utilize dietary energy when fat prices are relatively high and added energy levels in the diets are low. However, a decline in the cost of soybean oil might mean supplemental fat becomes cost effective as an energy source in pig diets and there is not much added benefit to feeding narasin.

This same cost-benefit analysis must also be considered when nutritionists evaluate the inclusion of additional feed ingredients to dietary formulations. Narsin has been shown to improve ADG and G:F. Rickard et al. (2017) reported that feeding 15 mg/kg narasin to finishing pigs improved ADG by 1.1%, but did not affect ADFI or G:F. Arkfled et al. (2015) reported a 2.0% improvement in ADG in barrows fed narasin, but not in gilts. In the current study, pigs fed 4% added fat and 15 mg/kg narasin improved ADG by only 0.9% compared to pigs fed 4% added fat and 0 mg/kg narasin (*P* = 1.00), suggesting that the additive effects of narasin are diminished as fat (energy) was increased. Both Rickard et al. (2017) and the current study used mixed-sex pens. Arkfeld et al. (2015) used split-sex pens and reported higher feed intakes in barrows compared to gilts and therefore potentially ingested a greater volume of narasin resulting in a difference in response. Agostini et al. (2014) reported that pigs housed in split-sex pens had lower total feed intake and better feed conversion than pigs in mixed-sex pens. Raising pigs in mixed-sex pens can increase variation and may mask the benefits of narsin.

Historically, there has been an inconsistent response between added fat and ADG (Engel et al., 2001; Bromm et al., 2023). In the current trial, there was no response on ADG to added fat when no narasin was added to the diet. However, there was a linear increase in ADG with increasing levels of fat when 15 mg/kg narasin was added to the diet. The reason for this interaction is not clear. Carcass data were not collected in the current study, which would help potentially clarify differences in lean tissue deposition among treatments. De la Llata et al. (2001) reported that ADG improved linearly as pigs were fed increased levels of added fat. The 4% added fat diet in De la Llata et al. (2001) contained 3,523 kcal/kg ME and led to a 5.7% increase in ADG compared to pigs fed 0% added fat (3,350 kcal/kg ME). This means that pigs in De la Llata et al. (2001) required approximately 30 kcal of energy to increase growth by 1%. To compare, pigs fed 4% added fat in the current study improved ADG by only 1.9% relative to pigs fed 0% added fat and needed approximately 111 kcal to increase growth by 1%. This could partly be attributed to the relatively high performance of pigs in the current trial. Pigs fed 0% fat in De la Llata et al. (2001) had an ADG of only 0.70 kg/d. Pigs today generally perform better (i.e., have greater ADG) than pigs in 2001 as genetic improvement has continued. De la Llata et al. (2001) also reported that feeding 6% added fat (3,610 kcal/kg ME) increased ADG by 6.1% compared to 0% added fat but only 0.4% compared to pigs fed 4% added fat, suggesting a diminishing marginal return to added fat in regard to ADG. Diets in the current study had overall less energy compared to previous research De la Llata et al. (2001) and Stephenson et al. (2016) and the response to energy did not seem to reach a point of diminishing returns.

Stephenson et al. (2016) reported that pigs fed 4% added fat (3,528 kcal/kg ME) tended to gain 3.6% more kg per day compared to pigs fed 0% added fat (3322 kcal/kg ME). Pigs in Stephenson et al. (2016) needed approximately 57 kcal ME to increase growth by 1%. Pigs fed 0% added fat in Stephenson et al. (2016) had an ADG of 1.06 kg/d. It is also worth noting that Stephenson et al. (2016) was conducted in small pens with only 2 pigs per pen and provided 50% more space per pig than the current trial. Feed intake has been shown to decrease as stocking density increases (Gonyou and Stricklin, 1998). It is thought that increasing dietary energy could have a differing effect on growth performance depending on space availability, which potentially could explain the discrepancy on ADG with the current trial.

De la Llata et al. (2001) reported feed efficiency improved by 8.8% for pigs fed 4% added fat compared to pigs fed no added fat (De la Llata et al., 2001), a gain of approximately 2% for every 1% increase in added fat. The pigs in the current study responded very similarly with an increase in G:F of 7.9% for pigs fed 4% added fat compared to 0% added fat. Pigs in the current study required approximately 27 kcal ME of energy to increase G:F by 1% and pigs in De la Llata et al. (2001) required approximately 20 kcal ME to increase G:F by 1%. De la Llata et al. (2001) also reported that pigs fed 6% added fat increased G:F by 4.1% compared to pigs fed 4% added fat, indicating that adding 6% added fat to a 3350 kcal/kg ME basal energy diet was still beneficial for feed efficiency. Additionally, the interaction between narasin and energy in the current study becomes more pronounced during the later dietary periods. This trend is consistent with previous research, where the effects of narasin increases as G:F decreases (Becker et al., 2024)

Overall, increasing dietary energy by increasing fat improved gain:feed regardless of narasin inclusion. However, adding narasin at 15 mg/kg improved gain:feed by 2.6% with 0% added fat but provided no additional benefit when fed with 4% added fat. The additive benefits of feeding narasin diminished as fat level increased. Both supplemental fat and narasin can improve growth performance of pigs, but as performance continues to improve and pigs approach their genetic potential for growth, marginal benefits in improvement become increasingly difficult. It may not be beneficial to include both additional fat and narasin in finishing pig diets at the same time to growing-finishing pigs as there was a diminishing improvement to narasin as fat level increased.

## Supplementary Data

Supplementary data are available at *Translational Animal Science* online.

txaf088_suppl_Supplementary_Figure_1

txaf088_suppl_Supplementary_Figure_2

txaf088_suppl_Supplementary_Figure_3
