# Supplementary figures and images for: The effects of feeding increasing levels of added fat to growing-finishing pigs when fed with or without narasin (Skycis)
Source: Transl Anim Sci. 2025 Jul 12;9:txaf088. doi: 10.1093/tas/txaf088 (PMC12607923; doi:10.1093/tas/txaf088)

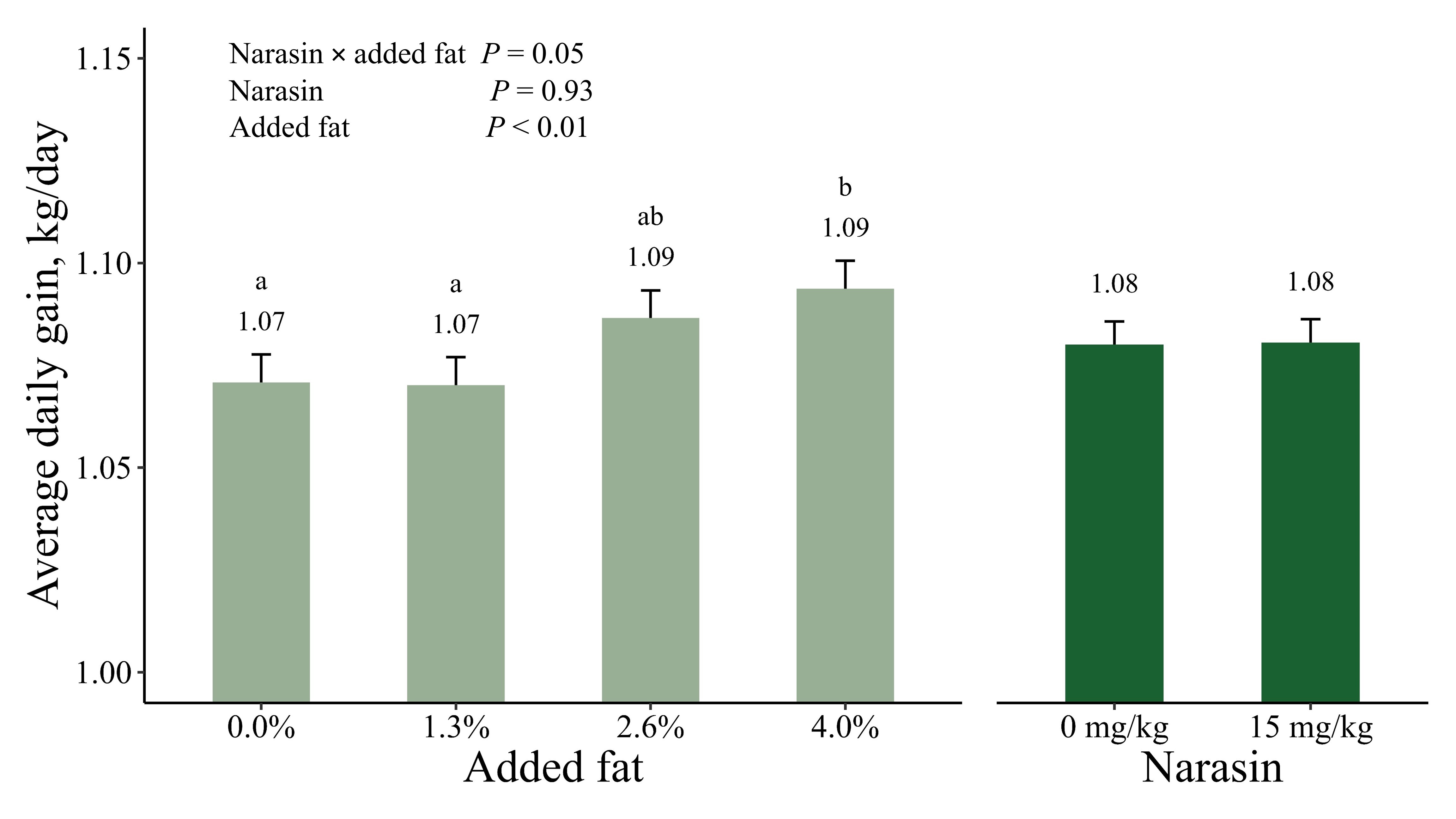

Supplement: txaf088_suppl_Supplementary_Figure_1 [file txaf088_suppl_supplementary_figure_1.jpeg]

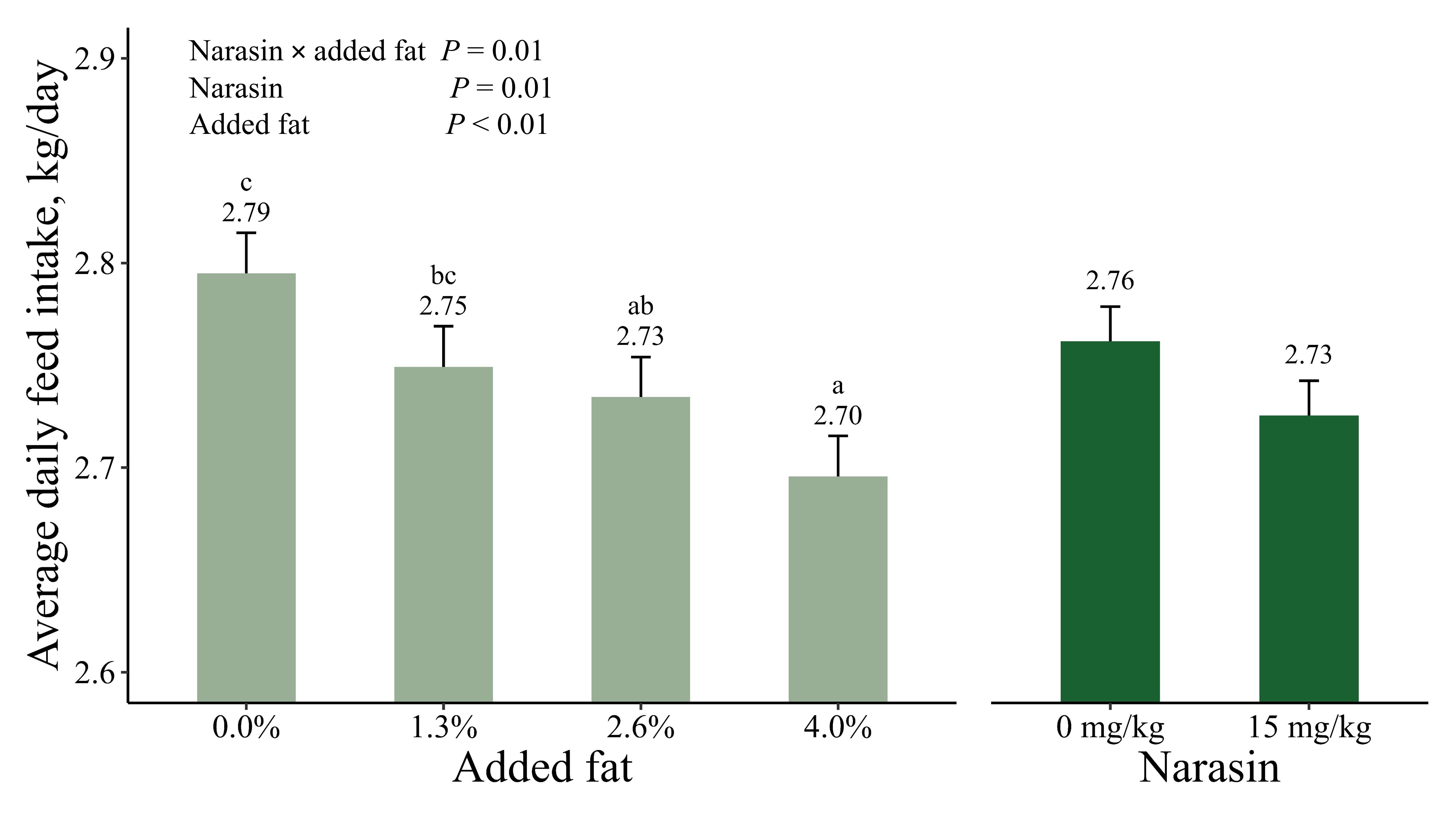

Supplement: txaf088_suppl_Supplementary_Figure_2 [file txaf088_suppl_supplementary_figure_2.jpeg]

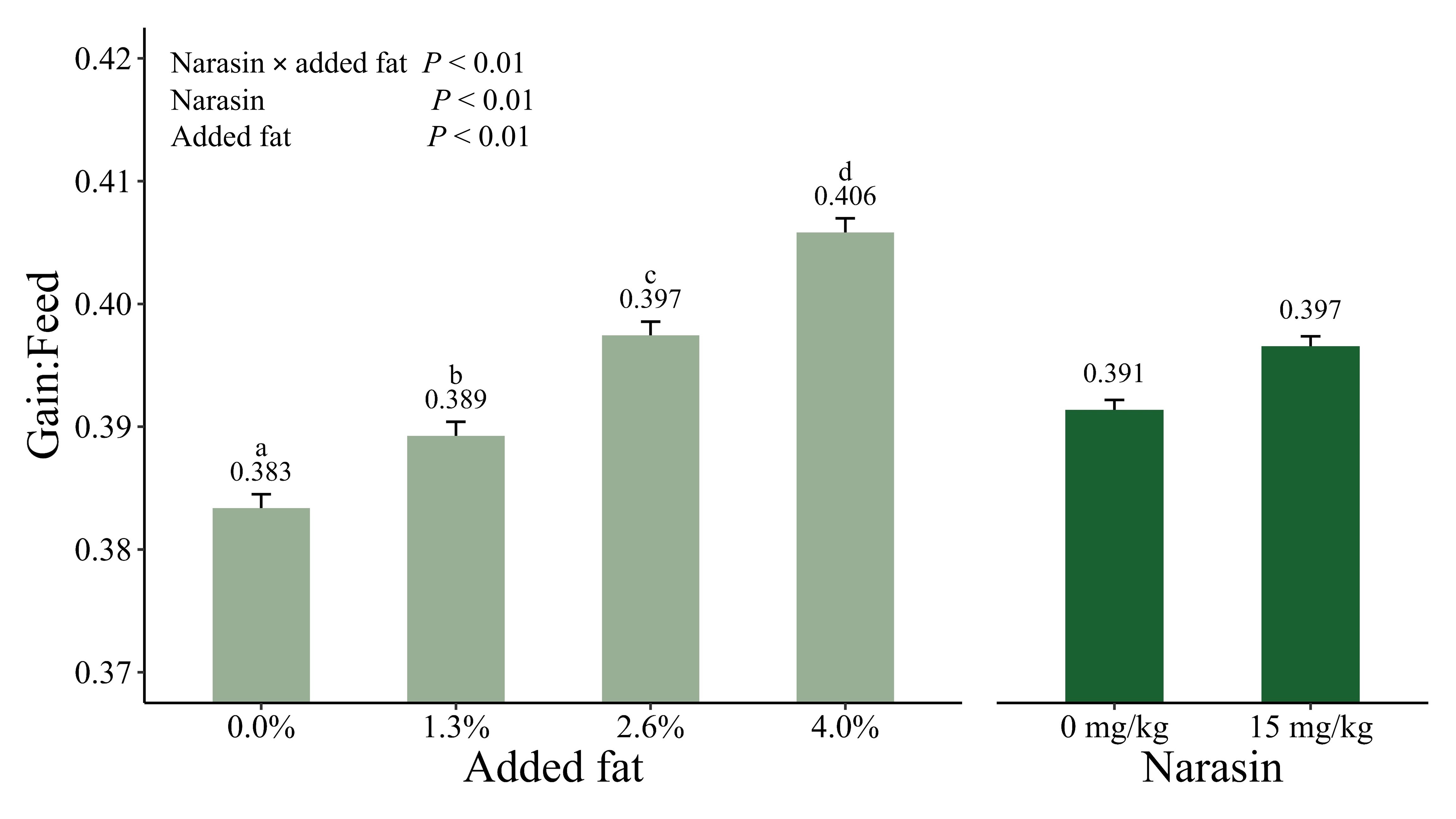

Supplement: txaf088_suppl_Supplementary_Figure_3 [file txaf088_suppl_supplementary_figure_3.jpeg]
